# Why marine phytoplankton calcify

**DOI:** 10.1126/sciadv.1501822

**Published:** 2016-07-13

**Authors:** Fanny M. Monteiro, Lennart T. Bach, Colin Brownlee, Paul Bown, Rosalind E. M. Rickaby, Alex J. Poulton, Toby Tyrrell, Luc Beaufort, Stephanie Dutkiewicz, Samantha Gibbs, Magdalena A. Gutowska, Renee Lee, Ulf Riebesell, Jeremy Young, Andy Ridgwell

**Affiliations:** 1 School of Geographical Sciences, University of Bristol, University Road, Bristol BS8 1SS, UK.; 2 GEOMAR Helmholtz Centre for Ocean Research Kiel, Düsternbrooker Weg 20, 24105 Kiel, Germany.; 3 Marine Biological Association, The Laboratory, Citadel Hill, Plymouth PL1 2PB, UK.; 4 Department of Earth Sciences, University College London, Gower Street, London WC1E 6BT, UK.; 5 Department of Earth Sciences, University of Oxford, South Parks Road, Oxford OX1 3AN, UK.; 6 Ocean Biogeochemistry and Ecosystems, National Oceanography Centre, Southampton SO14 3ZH, UK.; 7 Ocean and Earth Science, University of Southampton, Southampton SO17 1BJ, UK.; 8 Aix-Marseille University/CNRS, Centre Européen de Recherche et d’Enseignement des Géosciences de l’Environnement (CEREGE), 13545 Aix-en-Provence, France.; 9 Department of Earth, Atmospheric and Planetary Sciences, Massachusetts Institute of Technology, Cambridge, MA 02139, USA.; 10 Monterey Bay Aquarium Research Institute, 7700 Sandholdt Road, Moss Landing, CA 95039, USA.; 11 Museum of Natural History, Cromwell Road, London SW7 5BD, UK.; 12 Department of Earth Sciences, University of California, Riverside, Riverside, CA 92521, USA.

**Keywords:** Coccolithophores, calcification, photosynthesis, trade-offs, ecological and physiological costs and benefits, ecosystem modeling

## Abstract

Calcifying marine phytoplankton—coccolithophores— are some of the most successful yet enigmatic organisms in the ocean and are at risk from global change. To better understand how they will be affected, we need to know “why” coccolithophores calcify. We review coccolithophorid evolutionary history and cell biology as well as insights from recent experiments to provide a critical assessment of the costs and benefits of calcification. We conclude that calcification has high energy demands and that coccolithophores might have calcified initially to reduce grazing pressure but that additional benefits such as protection from photodamage and viral/bacterial attack further explain their high diversity and broad spectrum ecology. The cost-benefit aspect of these traits is illustrated by novel ecosystem modeling, although conclusive observations remain limited. In the future ocean, the trade-off between changing ecological and physiological costs of calcification and their benefits will ultimately decide how this important group is affected by ocean acidification and global warming.

## Teaser

Calcification in coccolithophores has high energy demand but brings multiple benefits enabling diversity of ecology and form.

## INTRODUCTION

An estimated 200 species of coccolithophores live in the modern ocean (*1*) across a wide spectrum of surface ocean environments, ranging from highly productive eutrophic waters in temperate and subpolar regions to the permanently oligotrophic waters of the subtropical gyres. Coccolithophores usually contribute to 1 to 10% of primary production and phytoplankton biomass in subpolar, temperate, and tropical environments (*2*), increasing to as much as 40% under bloom conditions (*3*). Alongside foraminifera, coccolithophores are the most productive pelagic calcifiers on the planet. They generate a continuous rain of calcium carbonate to the deep ocean, maintaining a vertical gradient in seawater alkalinity and thus being co-responsible for the carbonate pump (*4*). This coccolith rain has also helped create the largest geological sink for carbon, whereas the sensitivity of sea-floor carbonate accumulation to the carbon cycle gives rise to an important stabilizing feedback in Earth’s climate system (*5*). Furthermore, the dense mineral coccoliths provide ballast that facilitates effective transport of organic matter to the deep ocean (*6*), thereby potentially contributing to the vertical CO_2_ gradient in the ocean (*7*). The important contribution of coccolithophores in regulating ocean biogeochemical cycles and climate requires that we adequately understand their physiological and ecological functioning and response to changing conditions to be able to project future changes in biogeochemical cycles.

**Fig. 1:**
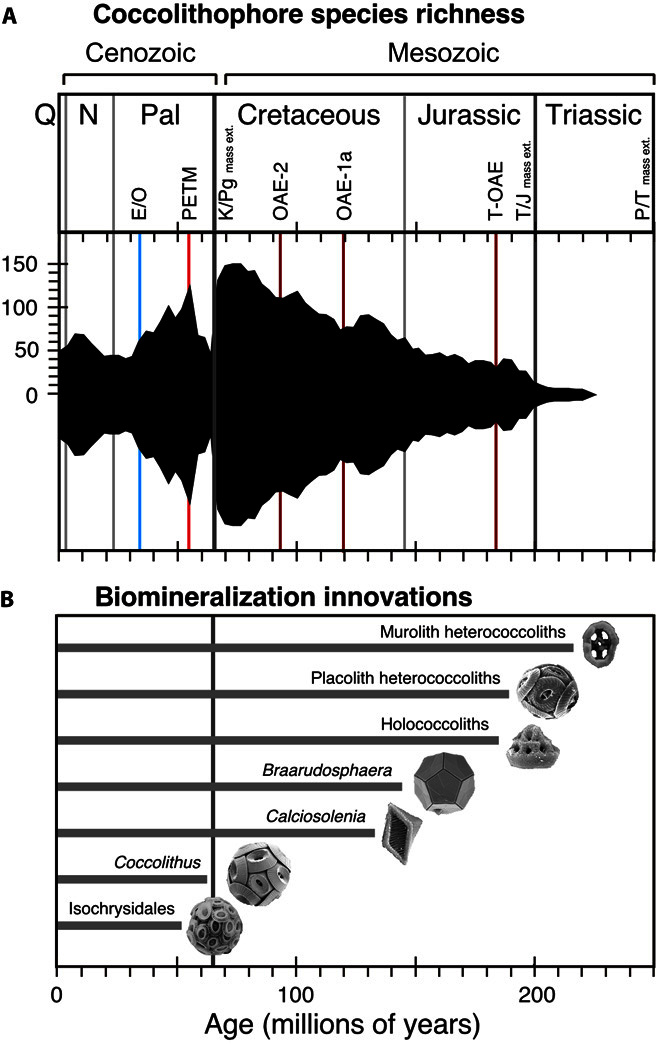
Evolutionary history of coccolithophores. (**A**) Coccolithophore species richness over time [combining heterococcoliths and nannoliths; data from Bown *et al.* (*10*)]. Q, Quaternary; N, Neogene; Pal, Paleogene; E/O, Eocene/Oligocene glacial onset event; PETM, Paleocene/Eocene thermal maximum warming event; K/Pg, Cretaceous/Paleogene; OAE, oceanic anoxic event; T-OAE, Toarcian oceanic anoxic event; T/J, Triassic/Jurassic; P/T, Permian/Triassic; mass ext., mass extinction. (**B**) The fossil record of major coccolithophore biomineralization innovations and morphogroups, including the first appearances of muroliths (simple coccoliths with narrow, wall-like rims), placoliths (coccoliths with broad shields that interlock to form strong coccospheres), holococcoliths (coccoliths formed from microcrystals in the haploid life cycle phase), *Braarudosphaera* (pentagonal, laminated nannoliths forming dodecahedral coccospheres); *Calciosolenia* (distinct, rhombic murolith coccoliths), *Coccolithus* (long-ranging and abundant Cenozoic genus), Isochrysidales (dominant order that includes *Emiliania*, *Gephyrocapsa*, and *Reticulofenestra*). Significant mass extinctions and paleoceanographic/paleoclimatic events are marked as horizontal lines.

Coccolithophores are characterized by the production of calcite platelets (coccoliths) that adorn the cell surface to form an exoskeleton (coccosphere). The fossil record of coccoliths stretches back to at least 209 million years ago (Ma), indicating the emergence of calcite biomineralization within the haptophyte algal group in the Late Triassic (Fig. 1). The origin of the haptophytes is far more ancient, with molecular genetic analysis placing their divergence from other algal groups within the Neoproterozoic, around 1200 Ma (*8*, *9*). Despite this long history of marine phytoplankton without mineralized coccoliths, the appearance of coccolithophores and the acquisition of calcite biomineralization marked the beginning of a near-unidirectional diversification trend and also the first significant deposition of carbonate on the open-ocean sea floor during Earth’s history. Estimates of coccolithophore diversity through time [for example, Bown *et al.* (*10*)] reflect the rapid accumulation of morphological innovation and variability in coccolith architecture and show the increase in species richness that characterized their early evolutionary history (Fig. 1). This trend was only interrupted by the singular and apparently instantaneous environmental perturbation associated with the Cretaceous-Paleogene boundary mass extinction event (66 Ma) (*11*), which eliminated more than 90% of coccolithophore species (*10*), and then again by the longer-term diversity decline, which accompanied the switch to ice-house climates through the Eocene and Oligocene (*12*). The overall trend of coccolithophore evolution over the past ~30 million years has been toward lower diversities with the progressive loss of species that produce large and heavily calcified coccoliths (Fig. 1). Although this trend has resulted in reduced coccolith sizes in today’s dominant species compared with older Paleogene and Cretaceous counterparts [for example, Bown *et al.* (*13*) and Hermann and Thierstein (*14*)], the modern community has nevertheless retained a spectacularly diverse array of coccolith architectures and cell shapes.

**Plate 1:**
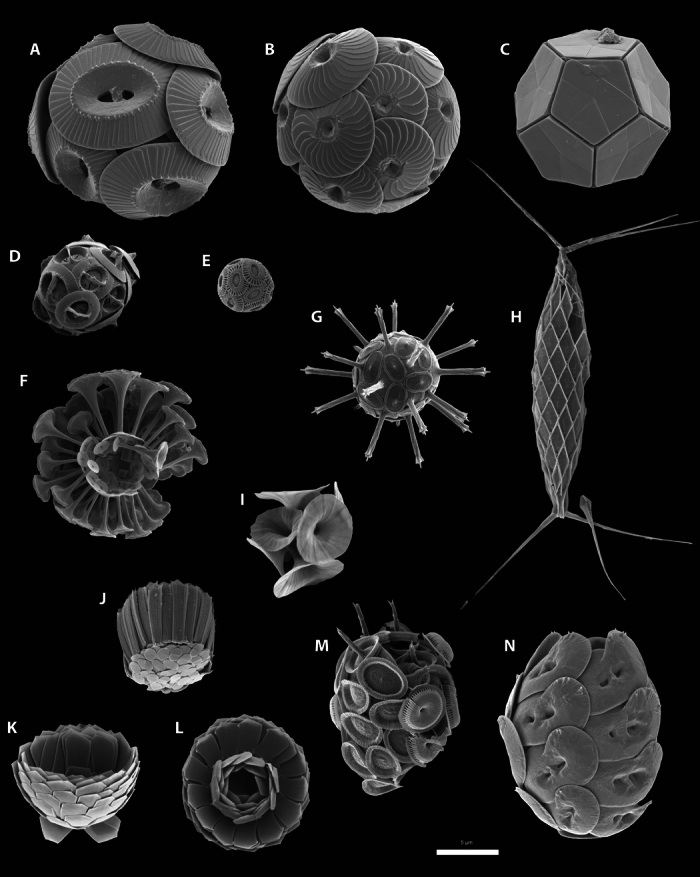
Diversity of coccolithophores. *Emiliania huxleyi*, the reference species for coccolithophore studies, is contrasted with a range of other species spanning the biodiversity of modern coccolithophores. All images are scanning electron micrographs of cells collected by seawater filtration from the open ocean. (**A** to **N**) Species illustrated: (A) *Coccolithus pelagicus*, (B) *Calcidiscus leptoporus*, (C) *Braarudosphaera bigelowii*, (D) *Gephyrocapsa oceanica*, (E) *E. huxleyi*, (F) *Discosphaera tubifera*, (G) *Rhabdosphaera clavigera*, (H) *Calciosolenia murrayi*, (I) *Umbellosphaera irregularis*, (J) *Gladiolithus flabellatus*, (K and L) *Florisphaera profunda*, (M) *Syracosphaera pulchra*, and (N) *Helicosphaera carteri*. Scale bar, 5 μm.

Morphologically, all coccolithophores share the same basic body plan of a cell surrounded by the exoskeletal coccosphere, but there is a marked variability in the shape of the cell; the shape, architecture, and crystallography of coccoliths; and their number, diversity, and arrangement around the cell (Plate 1). Coccosphere shapes range from spherical to cylindrical, with sizes ranging from ~3 to 30 μm. The number of coccoliths per coccosphere varies from as few as six to several hundred, in either single or multiple layers, whereas coccoliths themselves range from simple disk-like shapes to those with elaborate ornamentation or protrusions including long spines, trumpet-shaped projections, and delicate grills (Plate 1). Furthermore, many species only produce a single type of coccolith, whereas the coccosphere is made up of several types of coccolith in others. Finally, there is variation in the coccolith biomineralization mode depending on the phase of their haploid-diploid life cycle in which they are produced (*15*). During the diploid phase, coccolithophores produce heterococcoliths, formed from a radial array of large, complexly intergrown calcite crystal units. By contrast, in the haploid phase, many species produce holococcoliths, which are formed from minute (~0.1 μm), equidimensional calcite rhombohedra held together by an organic matrix (*16*). Heterococcolith and holococcolith biomineralization originated in the initial early Mesozoic diversification of coccolithophores, and the different cell shapes, the various coccolith types, and the diversity in architecture are also conservative features of coccolithophore biology that we are now able to identify through millions of years of their biomineralization history (Fig. 1) (*17*). The combined effect of this variability in the shape and size of coccoliths, their arrangement in the coccosphere, and the shape and size of the coccosphere produces remarkable morphological diversity within the group (Plate 1).

**Fig. 2:**
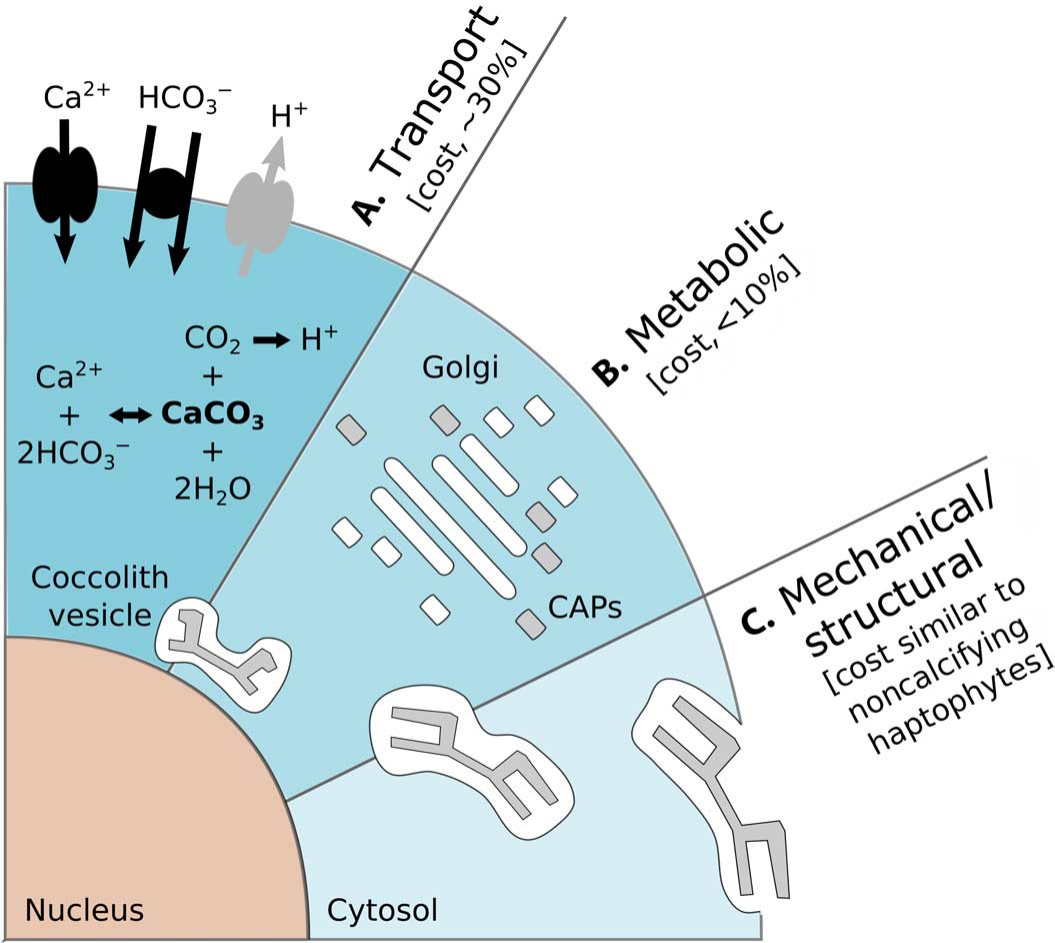
Schematic of the cellular processes associated with calcification and the approximate energetic costs of a coccolithophore cell. Energetic costs are reported in percentage of total photosynthetic budget. (**A**) Transport processes include the transport into the cell from the surrounding seawater of primary calcification substrates Ca^2+^ and HCO_3_^−^ (black arrows) and the removal of the end product H^+^ from the cell (gray arrow). The transport of Ca^2+^ through the cytoplasm to the CV is the dominant cost associated with calcification (Table 1). (**B**) Metabolic processes include the synthesis of CAPs (gray rectangles) by the Golgi complex (white rectangles) that regulate the nucleation and geometry of CaCO_3_ crystals. The completed coccolith (gray plate) is a complex structure of intricately arranged CAPs and CaCO_3_ crystals. (**C**) Mechanical and structural processes account for the secretion of the completed coccoliths that are transported from their original position adjacent to the nucleus to the cell periphery, where they are transferred to the surface of the cell. The costs associated with these processes are likely to be comparable to organic-scale exocytosis in noncalcifying haptophyte algae.

Such diversity of form and long-term conservatism of morphological features in coccolithophores prompt the question of what the underlying advantages of biomineralization are. In broadest terms, the production of mineralized plates is likely to be the coccolithophorid solution to the need to produce a protective cell covering, a challenge imposed on multiple plankton groups such as diatoms, which form siliceous skeletons, and dinoflagellates, which use both calcium carbonate and toughened intracellular organic plates. However, beyond this general need for a protective covering, there is also likely to be a more sophisticated function arising from the coccosphere morphology, as evidenced by the broad biogeographical associations between types of environment and characteristic coccosphere and coccolith architectures (*18*). For instance, as noted by Young (*18*), oligotrophic gyres tend to be characterized by *U. irregularis* and *D. tubifera* (Plate 1 and fig. S1), which are both nonmotile coccolithophores with large, low-density coccospheres formed from coccoliths with large, trumpet-shaped structures around much smaller organic cells. Mesotrophic and eutrophic environments are dominated in abudance by placolith-bearing coccolithophores such as genus *Gephyrocapsa* (including *E. huxleyi*), *C. pelagicus*, and *C. leptoporus*, which all have robust and interlocking coccospheres made up of flattened, disc-shaped “placolith” coccoliths (fig. S1). Deep subeuphotic environments are dominated in biomass and calcite production by *F. profunda* and *G. flabellatus,* both of which are motile species with relatively small coccospheres made up of distinctive scales and blade-like coccoliths (Plate 1).

The occurrence of specific coccolithophore biogeographical assemblages with distinct coccosphere architectures hints at an underlying link between coccolith formation and ecological adaptation. Although there is good understanding on coccolithophore ecology in terms of the environmental controls on the distribution and response to environmental changes of *E. huxleyi* (*19*–*23*), the intriguing degree of intricacy and variety of coccolith forms still fuels the ongoing debate as to why coccolithophores calcify. In the remainder of this paper, we assess current evidence for the costs and benefits of calcification to address the central question of “why” coccolithophores calcify and why they do so with such diversity of form. A better understanding of the role of calcification allows us to further address the potential vulnerability of this key phytoplankton group to future global change.

## COSTS OF CALCIFICATION

The biomineralization of calcitic CaCO_3_ in the form of coccoliths is an extraordinary physiological feature. The rates of substrate transport in coccolithophores are among the greatest ion fluxes reported in eukaryotic organisms. Coccolithophores produce massive quantities of calcite (~1 to 2 coccoliths per hour), equivalent in carbon units to producing their entire organic cell mass on a daily basis. This is accompanied by the cellular challenge of a large secretion event every time a newly biomineralized coccolith is transferred out of the cell and arranged in the coccosphere.

Formation of coccoliths takes place in a Golgi-derived vesicle termed “coccolith vesicle” (CV; Fig. 2). Within the CV, coccolith-associated polysaccharides (CAPs) are thought to regulate the crystal nucleation of calcite (CaCO_3_) and its subsequent growth (*24*). The nucleation of CaCO_3_ is typically initialized around the rim of a preformed organic baseplate, and the crystal growth is then regulated through interactions of intercrystalline and intracrystalline CAPs and a protein matrix (*25*). When CaCO_3_ nucleation is finished, CAPs remain on the surface of the coccolith, thereby encasing it within an organic coating. The completed coccolith subsequently migrates to the outer region of the cell, where the CV merges with the cell membrane and releases the coccolith to the cell surface (*26*, *27*). We estimate here the different potential ecological and physiological costs associated with calcification in coccolithophores, including energetic costs, the impact of carbonate chemistry, nutrient costs, and the effect of higher sinking rate.

### Energetic costs

The energetic costs of calcification can be categorized into costs associated with the following: delivery and removal of key substrates and products to or from the CV and cytosol, such as Ca^2+^, HCO_3_^−^, and H^+^ (transport costs); production of associated organic materials such as polysaccharides (metabolic costs); secretion of mature coccoliths (mechanical costs); and construction and maintenance of additional cytoskeletal and other structural components needed for coccolithogenesis (structural costs) (Fig. 2). Transport of ionic substrates or products against their electrochemical potential gradients across either the plasma membrane or the intracellular calcifying compartment membrane is driven either directly via chemical energy supply to ion pumps or indirectly by using the electrochemical potential gradient of another ion, itself established by membrane pumps. So long as the transport pathways, fluxes, and concentrations of particular ions in relevant compartments are known, a transport energy budget can be estimated.

While the exact transport pathway for delivery of Ca^2+^ to the CV has yet to be confirmed, current evidence strongly suggests a channel-mediated entry of Ca^2+^ across the plasma membrane with an endomembrane-localized active transport, such as the activity of a Ca^2+^/H^+^ antiporter (*28*, *29*). The energetics of Ca^2+^ transport is thus likely to be significantly determined by the constrained nature of the Ca^2+^ transport pathway and the need to keep Ca^2+^ concentrations in the cytosol at a minimum to avoid toxicity in the cell. A significant assumption in determining the cost of delivery of Ca^2+^ is the required amount of Ca^2+^ in the calcifying compartment, which is determined, in turn, by the saturation state necessary for calcite precipitation (Ω = [Ca^2+^][CO_3_^2−^]/*K*_sp_ > 1, where *K*_sp_ is the solubility constant). A simple scenario of intra-CV inorganic carbon and pH values close to seawater concentrations gives estimates of the energy required to raise the concentration of Ca^2+^ to achieve calcite precipitation between 4.5 and 30 kJ/mol (*30*). Thus, the upper value of Ca^2+^ transport cost represents as much as 20% of the equivalent cost of fixing 1 mol of organic carbon by photosynthesis (Table 1). For HCO_3_^−^ transport into and subsequent H^+^ removal out of the CV, the solubility product *K*_sp_ determines again the amount of CO_3_^2−^ required for calcite precipitation (and its energetic cost). HCO_3_^−^ transport costs can be estimated from assumed cellular concentrations by calculating the transmembrane electrochemical potential gradients for HCO_3_^−^. Assuming a net 10-fold accumulation of HCO_3_^−^ above the external seawater concentration as observed in the cell of *E. huxleyi* (*31*) and a membrane potential of −50 mV (*32*), the electrochemical potential gradient for HCO_3_^−^ will require the energy equivalent of approximately 0.2 ATP per mole of HCO_3_^−^. Assuming that 1 mol of HCO_3_^−^ produces 1 mol of CO_3_^2−^, then the cost of HCO_3_^−^ transport for calcification is approximately 5% of the energy requirement for organic carbon fixation for a cell calcifying with a calcification/photosynthesis ratio of 1. For H^+^ removal costs, a current hypothesis based on the observation of strong up-regulation of H^+^/Ca^2+^ antiporters in calcifying cells of *E. huxleyi* (*29*) proposes a separation of Ca^2+^ accumulation into a CV precursor compartment, which is driven by the inside-acid H^+^ electrochemical gradient, and eventual alkalinization of the calcifying compartment. Earlier estimates of the cost of removing H^+^ from the CV precursor compartment during HCO_3_^−^ transport suggest an energetic cost equivalent to around 5% of the energy requirement for organic carbon fixation (*30*). These considerations therefore put the combined transport costs for Ca^2+^, HCO_3_^−^, and H^+^ at around 30% of the total photosynthetic energetic budget but vary with species, pH, and the degree of calcification (Table 1). This analysis compares to the recent estimate made by Raven and Crawfurd (*23*), who estimated calcification-related ion transport to cost 19% of the total photosynthetic energetic budget.

**Table 1:** Percentage of the total photosynthetic energy budget dedicated to components of calcification. The budget is presented for two main coccolithophore species (*E. huxleyi* and *C. pelagicus*). PIC, particulate inorganic carbon.

**Process**	***E. huxleyi***	***C. pelagicus***
Ca^2+^ transport	3% (CV pH of 8) to 20% (CV pH of 7.5)*	≫20%^†^
HCO_3_^−^ transport	5%^‡^	Undocumented but expected to be significant to sustain high PIC production rate
H^+^ (removal) transport	<5%^§^	5%*
Polysaccharide generation	7%	0.2%
Total	20–37%	≫25%

*Measured by Anning *et al.* (*30*).

†Estimated from *E. huxleyi*, assuming a 10-fold higher PIC production rate.

‡Because there is no direct measurement of HCO_3_^−^ accumulation in the cytoplasm, we used measurement of total cellular dissolved inorganic carbon (DIC) by Sekino and Shiraiwa (*31*), which is equivalent to a 10-fold accumulation. Following the electrochemical potential gradient equation for HCO_3_^−^, ΔμHCO_3_^−^ = *RT*ln*Co*/*Ci* + *zFV* (in kilojoules per mole), where Δμ is the electrochemical potential gradient, *R* is the gas constant, *F* is the Faraday constant, *z* is the valency, *T* is the temperature, *Co* and *Ci* are the external and internal concentrations of HCO_3_^−^, and *V* is the membrane potential (measured at −50 mV); a 10-fold HCO_3_^−^ concentration gradient across the membrane corresponds to ΔμHCO_3_^−^ ~ 10 kJ/mol. Considering that 1 mol of adenosine triphosphate (ATP) provides ~ 50 kJ per mole of energy for transport, moving 1 mol of HCO_3_^−^ against its electrochemical potential gradient then requires 0.2 ATP. Assuming a requirement of 3.2 ATP per mole for CO_2_ fixation and that 1 mol of transported HCO_3_^−^ produces 1 mol of CO_3_^2−^ and a 1:1 calcification/photosynthesis ratio, the cost of HCO_3_^−^ transport in terms of ATP required to fix 1 mol of CO_2_ by photosynthesis is thus equal to 0.2/3.2 ~ 5%.

§Estimated from *C. pelagicus*, assuming a lower PIC production rate, resulting in lower generation of H^+^.

Previous estimates suggest that the production of CAPs represents the dominant metabolic cost associated with calcification, where up to 50% of the energy requirements of organic carbon fixation is used to produce CAPs (*33*, *34*). However, these estimates, based on the hypothesis that Ca^2+^ transport to the site of calcification is achieved by polysaccharide binding, are derived from *Pleurochrysis carterae*, a coastal-dwelling coccolithophore, which uses three different CAPs to facilitate calcification. Other coccolithophore species produce fewer acidic polysaccharides. Here, we provide new estimates of the metabolic costs associated with polysaccharide generation in three common open-ocean coccolithophore species, each of which uses only one CAP for calcification (*35*). Our calculations, based on total CAP extracted from the average number of coccoliths per cell, suggest a much smaller proportional energetic cost (Table 1). *E. huxleyi*, *G. oceanica*, *C. braarudii* yield ~0.047, ~0.019, and ~0.034 pg of CAP per coccolith, respectively. Assuming coccolith production rates of 1 and ^1^/_3_ coccolith per hour for the Isochrysidales group (*19*) and *C. braarudii* (*27*), respectively, and net carbon fixation rates of 0.69, 0.58, and 6.18 pg of POC (particulate organic carbon) per hour for *E. huxleyi*, *G. oceanica* (*36*), and *C. braarudii* (*37*), respectively, we find much smaller metabolic costs in these species. The costs for the generation of polysaccharides that promote matrix-assisted nucleation (expressed in CAP per POC) range from 0.2% (*C. braarudii*) to 7% (*E. huxleyi*) of the total photosynthetic cost (Table 1).

Other mechanical and structural costs associated with calcification, such as cytoskeletal and associated machinery for secretion of coccoliths and associated energetic requirements, are difficult to quantify but are already an integral part of the physiology of haptophytes, all of which generate and exocytose organic scales. Therefore, these other unquantified costs are not directly part of the cost of calcification. On the basis of our analysis, Ca^2+^ transport is thus the dominant cost for calcification. A trend is also observed for the larger, more heavily calcified, and more ancient species (for example, *C. pelagicus*) to channel a greater proportion of their photosynthetic energy to calcification (Table 1). Given the range of uncertainties, calcification in coccolithophores is a sink for energy that is equivalent to approximately one-third of the total photosynthetic energetic budget but likely scales with the degree of calcification of the species.

### Impact of carbonate chemistry

Changes in ocean carbonate chemistry may affect the energetic cost of calcification-associated uptake of inorganic carbon and removal of H^+^ across the plasma membrane. For H^+^ extrusion under current conditions (seawater pH of 8.2; cytosolic pH of 7.3), H^+^ is close to equilibrium at measured membrane potentials around −50 mV (*32*). Therefore, the H^+^ electrochemical potential gradient (ΔμH^+^), represented by the sum of the membrane potential and the pH gradient (ΔμH^+^ = 2.3030*RT*ΔpH + *zFV*, where *RT* and *F* have their usual values, *z* is the valency, ΔpH is the pH gradient across the plasma membrane, and *V* is the membrane potential), is close to 0, requiring little or no energy for H^+^ removal. At future predicted decreased ocean pH, assuming constant cytoplasmic pH and membrane potential, H^+^ will need to be extruded against an electrochemical potential gradient. However, even at an assumed ocean pH of as low as 7.5, the H^+^ electrochemical potential gradient and consequent energy requirement for H^+^ extrusion would still be relatively small, equivalent to around 3% of the ATP requirement for photosynthetic carbon fixation. This relatively small extra energetic cost at low pH may be seen as surprising; laboratory experiments often show a large decrease in calcification rates under such conditions (*36*–*38*). This discrepancy could potentially be explained by H^+^ removal costs that are not considered in the calculation. Alternatively, high H^+^ concentration could exert a detrimental effect on the cell metabolism due to strong changes in intracellular pH, which can quickly follow changes in seawater pH, as shown for *E. huxleyi* (*39*). In particular, Taylor *et al.* (*32*) showed that the gating properties of the voltage-dependent H^+^ channel that provides the major route for H^+^ efflux at the plasma membrane are such that H^+^ efflux may be significantly compromised at lower external pH because the H^+^ channel tends to a closed state at lower external pH, consistent with its role in regulating pH in response to internal pH decreases.

### Nutrient costs

The requirements for the organic cellular components of a coccolithophore cell are similar to those for noncalcifying phytoplankton. In contrast, forming coccoliths need little other than inorganic carbon and calcium because CAPs have very low nitrogen and phosphorus requirements (*40*, *41*). From this perspective, coccoliths are “cheap” in terms of nutrient cost, which is supported by observations of continuing coccolith production when cell division ceases because of nutrient limitation (*42*–*44*).

### Sinking costs

The sinking rate of an organism increases with both the size and density of the cell as defined by Stokes’ law. The coccosphere thus influences the sinking rate of coccolithophores by making the cell both larger and denser (*45*), potentially causing coccolithophores to sink out of the euphotic zone before they can divide. We estimate here the sinking cost of calcification by comparing the effect of sinking rates between naked and calcified coccolithophores following the formation of Riley *et al.* (*46*), *D*_min_ = *v*^2^/[4 *g*(*I*_in_)], where *v* is the sinking velocity and *g*(*I*_in_) is the specific growth rate at incident light. Riley *et al.*’s formulation is based on an advection-diffusion vertical model and calculates the minimal turbulence of the mixed layer (*D*_min_) required to compensate for the sinking rate of an organism. If *D*_min_ is larger than the mixed-layer vertical mixing diffusivity, organisms sink out of the euphotic zone before reproducing. Observed vertical mixing diffusivity in the mixed layer is, on average, 1 × 10^−2^ m^2^ s^−1^, with values ranging between 3 × 10^−5^ and 1.5 m^2^ s^−1^, depending on the oceanic regions and time of sampling [for example, Fernández-Castro *et al.* (*47*)].

*E. huxleyi* is the smallest coccolithophore species (4 to 9 μm; Plate 1) and is omnipresent in all oceans except polar oceans. Laboratory experiments show that the coccosphere of *E. huxleyi* increases the sinking velocity by one order of magnitude, from ~3 to 30 cm day^−1^ for naked and calcifying cells, respectively (*45*). Using a specific growth rate of 0.7 day^−1^ (0.5 to 0.85 day^−1^) (*48*), we estimate *D*_min_ to be ~4 × 10^−9^ and ~4 × 10^−7^ m^2^ s^−1^ for naked and calcifying cells, respectively. Calcifying *D*_min_ is thus always lower than the observed values for mixed-layer vertical mixing diffusivity. Therefore, although there is a large impact of calcification on the sinking velocity, the impact of calcification on loss rates through sinking out of the mixed layer is negligible for *E. huxleyi*.

The situation is slightly different for larger cells of coccolithophores, for which calcification potentially causes the cell to sink out of the euphotic zone in weakly mixed upper ocean regions. We consider here the case of *Calcidiscus* spp., which is among the largest coccolithophore genera (12 to 20 μm; Plate 1) and is more abundant in mid- to low-latitude coastal communities and less abundant in temperate waters. Using estimates of calcifying *Calcidiscus* spp. sinking velocity of 4.3 m day^−1^ (for a 20-μm-diameter cell) (*45*) and a specific growth rate of 0.45 day^−1^ (0.36 to 0.54 day^−1^) (*48*), *D*_min_ is ~1.2 × 10^−4^ m^2^ s^−1^. The minimum turbulence required for calcifying *Calcidiscus* is thus smaller than most vertical eddy diffusivities observed in the mixed layer, except in regions with really low mixing, such as the South Atlantic subtropical gyre (*47*). To verify that this result is not only due to the larger size, we calculate the *D*_min_ of naked cells. The sinking velocity of naked *Calcidiscus* spp., estimated using Stokes’ law [*v* = 2/9 *g r*^2^ (ρ^cell^ − ρ^water^)/η^water^, where *g* is Earth’s gravitational acceleration (9.81 m s^−2^), *r* is a cell radius of 16 μm, ρ^cell^ is the cell density assumed to be the same as that of *E.huxleyi* (that is, 1090.6 kg m^−3^), ρ^water^ is the density of seawater (1025 kg m^−3^), and η^water^ is the dynamic viscosity of seawater (1.07 × 10^−3^ kg m^−1^ s^−1^) (*45*)], is ~0.7 m day^−1^. This results in a *D*_min_ of ~3 × 10^−6^ m^2^ s^−1^, which is smaller than the observed mixed-layer vertical diffusivity, such that the size itself does not account for the large calcifying *D*_min_. Thus, it is the possession of a coccosphere that makes it difficult for large cells to grow fast enough to outpace losses due to sinking in regions with very low turbulent mixing.

Our current knowledge of transport and metabolic processes underlying calcification indicates that, together, they potentially represent a significant energy sink with little, if any, nutrient costs. Our calculations also show that the coccosphere can add a sinking cost to large coccolithophores, preventing them from staying in the euphotic zone in weakly mixed environments. More work is needed on the mechanical and structural costs associated with calcification and the energy source allowing calcification to continue when photosynthesis shuts down under nutrient limitation, as well as on changes in intracellular pH and its effect on calcification under different carbonate conditions.

## BENEFITS OF CALCIFICATION

**Fig. 3:**
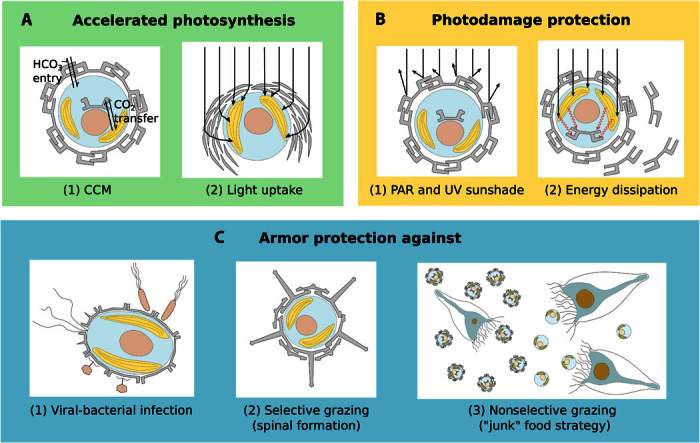
Proposed main benefits of calcification in coccolithophores. (**A**) Accelerated photosynthesis includes CCM (1) and enhanced light uptake via scattering of scarce photons for deep-dwelling species (2). (**B**) Protection from photodamage includes sunshade protection from ultraviolet (UV) light and photosynthetic active radiation (PAR) (1) and energy dissipation under high-light conditions (2). (**C**) Armor protection includes protection against viral/bacterial infections (1) and grazing by selective (2) and nonselective (3) grazers.

There has been wide speculation on the functions of calcification accrued by coccolithophores through the production and retention of coccoliths on the outside of the cell (*18*, *23*). An updated review of the main potential benefits of coccolithophore calcification is described in detail as follows and summarized in schematic in Fig. 3.

### Accelerated photosynthesis

It has been frequently suggested that calcification serves as a carbon concentrating mechanism (CCM) for photosynthesis because it reduces total alkalinity around the cell, thereby increasing the CO_2_ partial pressure (*P*co_2_) (either directly via CO_2_ supply or indirectly via H^+^ expulsion). This hypothesis has stimulated significant research effort in the past with some studies supporting the CCM idea (*30*, *49*, *50*), whereas others could not confirm it (*51*–*55*). Considering all experimental evidence, it seems most likely that calcification does not serve as the prime CCM for photosynthesis. This is supported by observations that most species cease calcification upon completing a single layer of abutting or overlapping coccoliths (*57*). Instead, calcification is likely to compete with photosynthesis for carbon supply from a common internal carbon pool (*56*, *58*). For instance, under extremely limiting conditions of DIC availability, *E. huxleyi* stops calcifying but continues to photosynthesize and divide at similar rates (*29*, *56*). Although this similarity in rates is consistent with a decoupling between calcification and photosynthesis, it also potentially indicates a benefit of photosynthesis that approximately counterbalances the energetic cost of calcification.

Another way in which calcification could promote photosynthesis is if coccoliths, which scatter light, do so in such a way as to funnel photons into the cell, increasing light availability to the chloroplasts and, therefore, photosynthesis (*18*). There is abundant evidence that coccoliths scatter light (*43*, *59*, *60*) in a manner dependent on the orientation of the coccoliths with respect to the incident photons (*61*). Cells living in the deep euphotic zone (<1% surface irradiance) are almost certainly light-limited rather than nutrient-limited. If coccoliths can be used to concentrate the little light available into the cell, calcification might benefit photosynthesis in low-light environments. Obvious candidates for testing whether calcification provides any tangible benefit in terms of light capture are *F. profunda* and *G. flabellatus* (Plate 1). These deep-dwelling coccolithophores are most numerous in low-light waters beneath the deep chlorophyll maximum, typically at depths of 50 to 150 m (*62*). Although the orientation of its coccosphere in the water column is not known, *F. profunda* organizes its coccoliths in a “radar dish” architecture (Plate 1). Calcification could thus potentially provide a particularly strong benefit to these deep-dwelling species, given that they synthesize relatively large amounts of calcite despite the energetic cost of calcification and that they live in a light-depleted environment. However, testing this possibility for *F. profunda* or *G. flabellatus* is hampered to date by the lack of success in keeping these species alive in the laboratory and the difficulty in observing them in the field.

### Protection from photodamage

Calcification might serve to protect the cell from photodamage (deterioration of photosynthetic performance due to damage from excess irradiance) for coccolithophore species living in the upper ocean. It might do so either by providing a sunshade (*63*, *64*) or as an energy dissipation mechanism under high-light conditions (*19*). Phytoplankton in general experience fluctuating light levels as they passively circulate through the depth of the mixed layer, facing a light difference of perhaps two orders of magnitude at the extreme between the surface of the mixed layer and its base. Along with additional variability in light availability due to the passing of clouds and the day-night cycle, this creates problems for the functioning and balanced metabolism of a phytoplanktonic cell.

Lohmann (*63*) first suggested that the coccosphere could potentially mitigate frequent radiative stress by protecting the cell as a sunshade, allowing the cell to tolerate high light levels. Observations of *E. huxleyi* show that the coccosphere may reduce PAR (400 to 700 nm) and UV (10 to 400 nm) transmission by about 10 to 20% (*65*). Very little is known about the influence of reduced light transmission on other coccolithophore species. However, for *E. huxleyi*, the sunshade effect for PAR is not thought to be critical because *E. huxleyi* is exceptionally resistant to photoinhibition even without a coccosphere (*66*–*68*). In contrast, the protection provided by the coccosphere to UV radiation appears beneficial even for high-light–adapted species such as *E. huxleyi*, as the absence of a coccosphere significantly reduces organic carbon fixation rates when cells in culture experience stressful UV radiation (*69*). A structural model study also shows that holococcoliths reflect more UV light while minimizing the loss of photosynthetically active light, by which the coccolith reduces the potential for cell photodamage (*70*). Therefore, for species inhabiting the upper part of the water column (the top 20 m in the clearest seawater), the coccosphere can presumably serve as UV protection.

Calcification could also benefit coccolithophores by providing them with an additional rapidly inducible energy sink under high-light conditions, preventing photodamage at little nutrient cost (*19*, *71*, *72*). Vast excess production of coccoliths is often observed in blooms of *E. huxleyi*, when many more coccoliths are produced than are required to complete a single covering of the cell, leading first of all to multiple layers of coccoliths around cells and finally to mass shedding of free coccoliths into the surrounding water (*19*, *42*, *73*). This is supported by laboratory experiments that show an up-regulation of calcification rates that is 10-fold stronger than that of organic carbon fixation rates after an abrupt light increase from 50 to 800 μmol photons m^−2^ s^−1^ (*71*), potentially suggesting a short-term energy dissipation function of calcification in coccolithophores.

### Hydrodynamic control

Phytoplankton living at the ocean surface are often nutrient-limited and could potentially benefit from sinking into nutrient-rich deeper waters. The ballast provided by the coccosphere accelerates the sinking rate of coccolithophores about 10-fold (see “Sinking costs” section), consistent with a hydrodynamic role for calcification in nutrient capture. In addition to the ballast effect, a higher degree of per-cell calcification (or PIC/POC ratio) usually coincides with increasing cell size, which further accelerates sinking velocities (see Materials and Methods). However, the gain of CaCO_3_ ballast–mediated movement seems to be trivial when compared to the substantial energetic costs associated with calcification. Even the very fast sinking coccolithophore species *C. leptoporus* only reaches sinking velocities of 4.3 m day^−1^ (*45*). Achieving a similar velocity by means of flagella movement would cost the cell much less than 1% of the total metabolic costs (*74*), with the additional benefit that the movement is not one-dimensional (1D) (downward) but could be directed toward a specific area of interest. From this, we conclude that calcification probably has little to do with control of the position in the water column.

### Armor protection against infections and grazing

Arguably, the most compelling hypothesis for the existence of the coccosphere is to provide an armor that protects the cell from predation, either by shielding against “penetrators” that enter and subsequently lyse the cell or by reducing, if not preventing, incorporation by “ingestors.”

Penetrators comprise a large variety of planktonic organisms from different functional groups. The smallest ones are viruses that can terminate blooms of *E. huxleyi* (*75*–*78*). To infect coccolithophores, viruses need to pass through the coccosphere to reach the cell membrane. In *E. huxleyi*, perforations within and between coccoliths are usually smaller than 200 nm and packed with polysaccharides so that coccoliths pose an effective barrier to viral infections. In vitro observations of viral attack have found viruses to detach immediately from *E. huxleyi* when blocked by the coccosphere (*79*). Another viral defense strategy has been identified in *E. huxleyi* where cells circumvent viral infection by switching from the diploid (calcified) to the haploid (only nonnoncalcified organic scales) life stage of *E. huxleyi* (*80*). This latter strategy is most likely not related to calcification per se but to metabolic and/or plasma membrane modifications of the host cell by which the virus becomes unable to recognize the haploid cell and fails to infect it. Although virus-like particles have been observed in cultures of a variety of coccolithophore species, nothing is currently known about whether other coccolithophore species are subject to viral infections. Evidence for viral shielding in coccolithophore species is therefore restricted by the viruses’ host specificity, together with the limited number of host-virus systems established so far. Other potential small penetrators of coccolithophores are infectious algicidal bacteria. Bacteria have very different life-styles from viruses and can be facultative infectious and not necessarily host-specific (*81*, *82*), allowing them to be a much more omnipresent threat even when the abundance of coccolithophores is low. As for viruses, perforations within coccoliths must be smaller than infectious bacteria to repel penetration, but no work on bacterial infections in coccolithophores has been published to date.

Microzooplankton (20 to 200 μm, usually dominated by protists) are probably the most potent grazers of coccolithophores because they typically account for two-thirds of the total grazing pressure in the ocean (*83*), and their optimal feeding size matches the size range of coccolithophores, which is 3 to 30 μm (*84*). Microzooplankton apply a variety of feeding strategies, including penetrating the cell with a feeding tube (peduncle) and subsequent suctioning of the organic matter [common in dinoflagellates (*85*)] or ingesting the whole prey. Ingestion by grazers that actively choose between prey organisms (selective grazing) can potentially be avoided by enlarging the coccosphere with modified, elongated, or spine-bearing coccoliths (Plate 1). Almost 50% of heterococcolith-bearing species described by Young *et al.* (*86*) apply such coccolith extensions, with some species even capable of extending them actively, presumably to frustrate the attacker (*87*). On the basis of kin selection (*88*), defense against nonselective grazers (for example, filter feeders) could be achieved indirectly by the large amount of calcareous “junk food,” which needs to be peeled off in a time- and energy-consuming process before reaching the valuable inner cell organics. Reducing the grazers’ growth by creating indigestion or prolonging digestion time translates to decreased grazing rates (*89*, *90*) and, consequently, increased net growth rates of the prey (*91*). This indirect defense mechanism can also be valuable for species with incomplete coccolith coverings, such as *F. profunda,* so that it may be one benefit of the coccosphere that applies to varying degrees for all species. Coccoliths thus represent non–energy-yielding material that must be ingested and processed alongside the organic matter, reducing the overall net nutritional value of coccolithophores and, hence, potentially reducing their desirability as prey.

Field and laboratory observations indicate that grazers discriminate against coccolithophores when other food sources are available (*92*–*97*). However, studies that compared direct grazing on calcified and noncalcified clones of the same coccolithophore species have shown that calcified cells are ingested slower than (*89*, *90*), at the same rate as, or faster than (*90*, *97*, *98*) noncalcified cells. The ambiguity of these results might come from effects independent of calcification such as the predator size selection (*97*), the type of grazers (*90*), the possibility of inducible defense mechanism in the haploid phase (*89*), the length of the experiment (*89*), and the decoupling between ingestion rate and growth rate (*90*). In particular, Harvey *et al.* (*90*) found that despite a 20% reduction in ingestion rate of the main heterotrophic dinoflagellate *Oxyrrhis marina*, their growth rate was still reduced by 66% when fed on calcified strains of *E. huxleyi*. Hence, although the coccoliths may have an important role in preventing and/or reducing grazing, additional clonal and longer experiments should be performed to disentangle the impact of grazing type (selective/generalist, size selection), the life cycle of coccolithophores, and the difference between ingestion and digestion rates on grazing protection.

The geological record supports the idea of an initial protective function for calcification, as coccolithophores appeared in the Triassic at virtually the same time as a second armored plankton group, the dinoflagellates, in the aftermath of the most severe mass extinction in the history of life, the end-Permian extinction (252 Ma) (*99*, *100*). The simultaneous appearance of these two armored plankton groups is strong evidence of a major reorganization within oceanic plankton. This also most likely reflects an increased predation pressure in the newly emergent marine ecosystems, which more broadly featured the appearance of novel and more effective predation that drove morphological and behavioral restructuring, in particular with the selection of infaunal modes of life and more effective defensive skeletons (*101*). Support for the critical and continued importance of protective functionality also comes from the observation that, once established, coccolith production has almost always been retained subsequently by coccolithophores, with rapidly increasing morphological diversity associated with all major evolutionary radiations and only one known example of secondary loss (Isochrysidaceae) (*10*, *102*).

## DISCUSSION AND PERSPECTIVES

Continuous fossil fuel CO_2_ emissions will induce a variety of environmental alterations in the ocean, with direct consequences for the marine ecosystem and planktonic organisms (*103*). For plankton confined to the sunlit surface ocean, such as coccolithophores, the most relevant future climate changes will be surface warming and ocean acidification. Rising sea surface temperature affects phytoplankton both directly through the temperature dependence of metabolic activities and indirectly through increased thermal stratification, leading to a reduced nutrient supply from deeper layers and enhanced average light levels due to the shoaling of the mixed layer (*104*). Changes in seawater chemistry associated with CO_2_-induced acidification could primarily affect coccolithophores in two ways: an increase in CO_2_ availability and an increase in hydrogen ion concentrations (decreased pH). The former alters photosynthetic carbon acquisition, whereas the latter can influence both calcification and photosynthesis of coccolithophores (*56*). Most of the culture studies performed on different species indicate that coccolithophore photosynthesis in some species is mildly stimulated and that cell division rate slightly decreased at elevated CO_2_/reduced pH (*105*–*107*). Because cell division rate is a key factor in determining fitness, the latter may put coccolithophores at a competitive disadvantage with acidification, although net population growth rates will be determined by relative mortality losses that more likely will be affected by climate change.

**Fig. 4:**
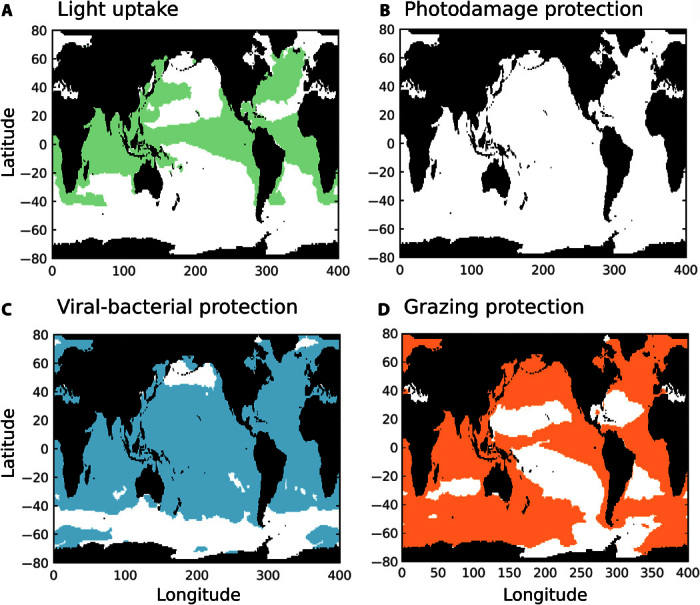
Potential niches of calcification benefits in coccolithophores using the MITgcm model. Model results show the geographical area of four tested benefits of calcification. (**A**) Benefit of light uptake (captured by increased photosynthesis-curve slope of the coccolithophore type). (**B**) Benefit of high-light protection (captured by reduced light inhibition of the coccolithophore type). (**C**) Benefit of protection against viral/bacterial infection (captured by reduced mortality rate of the coccolithophore type). (**D**) Benefit of grazing protection (captured in the model by reduced palatability of the coccolithophore type). Presented model results are from the most realistic simulations when compared with biomass observations along the AMT (fig. S3).

Whether these environmental changes in surface ocean conditions benefit or disadvantage coccolithophores depends on how they affect the fitness of coccolithophores in relation to the fitness of their main competitors and the nature of their predators. As an illustration of a way to disentangle the potential cost and benefits of calcification, a novel modeling approach is presented here (see Materials and Methods). This approach also links numerical models to explain oceanographic observations. The model used is the 3D MITgcm ocean plankton model of Dutkiewicz *et al.* (*108*), in which we also include a calcifying nanophytoplankton type (analogous to coccolithophores) in addition to a noncalcifying nanophytoplankton type (analogous to other haptophytes). To test hypotheses related to calcification, we impose a range of additional costs and benefits for the coccolithophore type. The energetic cost of calcification is imposed by reducing the maximum growth rate of coccolithophores relative to the noncalcifying types. For the benefits, four different possibilities are explored, including grazing protection (captured by reduced palatability of the calcifying types relative to the noncalcifying types), protection against viral/bacterial infection (reduced mortality), high-light protection (reduced photoinhibition), and light uptake (increased slope of the photosynthesis-irradiance curve). We compare the model results against field observations of coccolithophore and diatom biomass along the Atlantic Meridional Transect (AMT) (*109*) and statistically determine which combination of costs and benefits of calcification appears to be the most realistic (figs. S2 and S3). We explore a wide range of costs (10 to 90%) as well as a similar range of benefits and find that calcification is advantageous in distinct niches depending on the particular benefit (Fig. 4 and fig. S2). In particular, grazing protection appears to favor coccolithophores in (sub)polar, coastal, and equatorial areas (Fig. 4). These are the most eutrophic regions where grazing pressure is highest. Viral or bacterial protection appears to favor coccolithophores in most parts of the ocean except beyond 40°S and in the subpolar North Pacific Ocean, which might be related to temperature. Light uptake benefits favor coccolithophores in the equatorial regions, where they preferentially grow at the bottom of the mixed layer (50 to 100 m), and areas of the northern hemisphere where the mixed layer is deeper (around the Gulf Stream and Kuroshio currents). In these light-limited environments, the benefit of absorbing light better at low intensities provides a competitive edge relative to the noncalcifying types. Photodamage protection has a very limited effect in the model (see the Supplementary Materials for more information). Overall, the model results indicate that no single benefit can explain the distribution of coccolithophores (fig. S3). However, a combination of benefits in different ocean regions could lead to the model matching the observed biomass of diatoms and coccolithophores (fig. S3) (*108*), suggesting that there are multiple functions of calcification. In addition, we find that, depending on the type of benefit and environment, a range of associated energetic costs of calcification is possible in the model (10 to 50% of total energetic photosynthetic cost; see the Supplementary Materials). This suggests not only that there is a high physiological cost that is ecologically realistic when associated with an important benefit but also that the cost, and potentially the degree of calcification, can reflect the adaptation of coccolithophore species to their environment.

The potential for (and the likelihood of) multiple costs and benefits being involved in determining coccolithophore ecology raises a challenge on how to best draw conclusions from observations. For instance, the least calcified morphotypes of *E. huxleyi* and *Gephyrocapsa* were generally found in waters with the lowest CO_3_^2−^ concentration in one study (*110*), but in a second study (*111*), the most heavily calcified morphotypes of *E. huxleyi* were more abundant in the season with the most acidic (lowest saturation state) conditions. These examples suggest that appropriate care is needed in using spatial and temporal correlations between coccolith mass and environmental factors to predict the dominant controlling factors of calcification. However, the value of such observations might be enhanced by combining with ecological models that can be used to help untangle the different environmental influences on coccolithophore ecology and calcification.

This review of the history, physiology, and ecology of coccolithophores also incorporates new analysis of the energetic costs of calcification, as well as model-projected biogeographies driven by the nature of the assumed cost-benefit trade-off. We find that coccolithophore calcification is a highly demanding energy process, with the cost varying among species and with environmental conditions. Benefits associated with UV light and grazing protections have relatively well-supported evidence, whereas other potential benefits, such as light uptake and protection against viral/bacterial infection, are still very hypothetical. However, we conclude that although reduction in grazing pressure might have been the likely initial reason for why coccolithophores calcify, other benefits led to a substantial diversification in the different niches. The variability in calcification functions is consistent with the observed diversity and distribution of coccolithophores in the ocean, where placolith-bearing coccolithophores dominate in the subpolar regions (suggesting a function of grazing protection, depending on the location of light uptake and viral/bacterial protection), and *Umbellosphaera* and *Discosphaera* grow preferentially in the subtropical regions (suggesting mostly a function of viral/bacterial protection). Meanwhile, the haploid-diploid life cycle in coccolithophores is still poorly understood. The regular association of life stages with different biomineralization modes (typically heterococcoliths versus holococcoliths) also indicates a variability in the functions of calcification where the various coccolith morphologies produced within a single species during different life stages allow adaptation to different ecological niches (*67*, *80*, *112*–*115*). Because coccolithophores pursue a variety of growth strategies that allow them to flourish in waters ranging from oligotrophic recycling systems to eutrophic systems, their response to global change is likely to differ between members of the calcifying phytoplanktonic group. In particular, the numerically dominant coccolithophore species *E. huxleyi* may benefit from increased thermal stratification in the future relative to its competitors (*116*) because it is tolerant of high-light intensities (*19*) and has high affinities for phosphate uptake and utilization of organic phosphorus pools (*117*). Superimposed on this, coccolithophores may find that the increasing cost of calcification puts them at a relative disadvantage. The possibility of winners and losers among coccolithophore species in the future creates considerable challenges in projecting future marine ecosystem changes. We need more information regarding the physiological characteristics of a wide range of coccolithophore species differing in their likely ecological benefit for calcification and associated niche, together with an assessment of the trade-off between costs and benefits in a variety of oceanographic regimes, as well as the inclusion of this information in Earth system models.

## MATERIALS AND METHODS

### Model description

We used the 3D MITgcm physical ocean model (*118*) that was constrained with satellite and hydrographic observations (Estimation of the Circulation and Climate of the Ocean) (*119*) and combined with a plankton functional-type ecosystem based on the ocean biogeochemistry and ecosystem model of Dutkiewicz *et al.* (*108*). We contrasted the projected distribution of coccolithophores in this global ocean model against observations. The ecosystem model was based on five phytoplankton types (diatom, other large phytoplankton, *Prochlorococcus*, other small phytoplankton, and *Trichodesmium*-like diazotroph) and two zooplankton types (microzooplankton and mesozooplankton). Here, we added an intermediate size class of phytoplankton that represents a calcifying nanophytoplankton type (analogous to a coccolithophore) and a noncalcifying nanophytoplankton type (analogous to another haptophyte). We assumed that the noncalcifying nanophytoplankton type has averaged characteristics of “other large” and “other small” phytoplankton types (giving intermediate values for maximum growth rate, half-saturation constants, and light uptake) and an equal likelihood for grazing between microzooplankton and mesozooplankton. The calcifying type (coccolithophores) was given identical characteristics except for the cost and benefits described below.

To test hypotheses for calcification, we imposed on the modeled coccolithophore type additional costs and benefits relative to the noncalcifying nanophytoplankton. We accounted for the cost of calcification by reducing the maximum growth rate of the phytoplankton to capture the additional energy required for calcification. We did not investigate the impact of sinking cost because the model did not represent horizontally variable vertical diffusivity. For the benefit, we explored four different possibilities: light uptake (captured by increased photosynthesis-curve slope), photodamage protection (reduced photoinhibition), protection against viral/bacterial infection (reduced mortality), and grazing protection (reduced palatability). Because the overall costs and benefits of calcification could not be quantified a priori, a series of different values of costs and benefits were explored, covering the trade-off space of calcification for the four tested ecological benefits (fig. S2). A similar cost-benefit trade-off space experiment was used by Saito *et al.* (*120*) to explore the potential distribution of minimizing the need of iron in a nitrogen-fixing phytoplankton. This model design was shown to be useful in exploring the range of costs and subsequent reasonable benefits.

The model results showed that all hypothetical benefits can potentially be important for coccolithophores to survive in today’s ocean (blue area, fig. S2). However, the space of cost-benefit (or trade-off space) can vary, with a high range of success for benefits of light uptake and viral/bacterial protection and a narrower range of success for the grazing protection and photodamage protection benefits. Furthermore, not all survival strategies are realistic (see below). Some survival strategies are either too successful (for example, coccolithophores take over phytoplankton biomass on the global scale) or not successful enough (coccolithophores survive but at extremely low concentrations).

To determine the realistic space of trade-offs, we compared the model with observations of total biomass of coccolithophores and diatoms along the AMT (*109*). This data set was chosen because it has a large latitudinal spread (from equatorial to subpolar regions), and there was consistency of the measurement technique along the entire transect. This AMT shows that although diatom biomass peaks both at high latitudes (>40) and in the tropical North Atlantic (5°N to 20°N; around the Mauritanian upwelling), coccolithophore biomass varies by little more than an order of magnitude along the entire transect (~0.1 to 1 mg C m^−3^). We selected the model simulations that had realistic diatom/coccolithophore biomass in some portions of the AMT by calculating a cost function with χ^2^ statistics (*121*). Because phytoplankton biomass tends to be low, we took the log-transformed version of the χ^2^ fit to estimate the model-data comparison (the first equation in table S1). Finally, we followed Harmel and Smith (*122*) to take into account the uncertainties in the observations (the second equation in table S1). The results are presented in fig. S2 for the overall cost function and fig. S3 for the best model results.

### Coccolithophore sinking velocities in relation to degree of calcification, cellular density, and cell size

We examined sinking velocities, cellular densities, and cell size of the coccolithophores *E. huxleyi* (strain B92/11) and *G. oceanica* (strain RCC 1303) to investigate how a variable degree of per-cell calcification influences these three parameters. Variable calcification was achieved by culturing cells at different *P*co_2_ levels for *G. oceanica* and at different *P*co_2_ levels in combination with phosphorus limitation for *E. huxleyi*. *G. oceanica* cells were taken from 15° and 20°C experiments as described by Sett *et al.* (*123*). *E. huxleyi* was cultured as follows: Cells were grown in 2-liter dilute batch cultures in artificial seawater (*124*) at 15°C, at a photon flux density of 150 μmol m^−2^ s^−1^, and at a 16-hour light/8-hour dark cycle. *P*co_2_ (ranging from 180 to ~1000 μatm) was manipulated by adding variable amounts of NaHCO_3_, HCl, and NaOH. Artificial seawater was enriched with NaNO_3_ (9 μmol kg^−1^), Na_2_HPO_3_ (0.15 μmol kg^−1^), *f*/4 concentrations of a trace metal and vitamin mixture (*125*), SeO_2_ (10 nmol kg^−1^), and natural seawater (2 ml kg^−1^). The time when growth of the cells ceased (because of phosphorus limitation) was considered as the start of the stationary phase. Cells were then kept for three more days in the stationary phase in the culture bottles before being sampled for sinking velocity investigations (see below) or PIC and POC measurements [sampled and measured as described by Bach *et al.* (*38*)]. Sinking velocity was measured with the FlowCAM method developed by Bach *et al.* (*45*). Here, cells were carefully transferred in a settling chamber (inner dimensions: length, 43 mm; width, 3.6 mm; depth, 0.3 mm) with a pipette and filmed while sinking. The FlowCAM recorded the diameter of the cells, and the sinking velocity was calculated from changes in vertical position per time. The FlowCAM was placed in a temperature-controlled room (19°C), and the settling chamber was constantly ventilated with a fan to avoid convection. Furthermore, the low depth of the sinking chamber (0.3 mm) seemed to reduce turbulence (possibly due to capillary forces), because we never observed convection occurring in this setup [see Bach *et al.* for details (*45*)]. Cellular density was calculated by measuring sinking velocities and cell sizes, whereas known seawater density and viscosity were calculated using Stokes’ law (*45*).

Sinking velocities determined in this investigation were generally positively correlated with the PIC/POC ratio (fig. S4A), which was due to either the increase in coccosphere size (fig. S4B) or an elevated cellular density of the coccolithophores. Although a general positive trend is observed between cellular density and the PIC/POC ratio (fig. S4C), it should be noted that an increasing PIC/POC ratio does not necessarily lead to elevated cellular density (red triangles in fig. S4C) [see Hoffmann *et al.* (*126*)] and that accelerated sinking in case of a higher degree of per-cell calcification appears mostly to be caused by larger cell size (fig. S4B) instead of greater cellular density.

## Supplementary Material

http://advances.sciencemag.org/cgi/content/full/2/7/e1501822/DC1

## SUPPLEMENTARY MATERIALS

Supplementary material for this article is available at http://advances.sciencemag.org/cgi/content/full/2/7/e1501822/DC1 Supplementary Text table S1. Definition of the scores for the model-data comparison. fig. S1. Latitudinal biomass of two main coccolithophore types along the AMT. fig. S2. Testing of hypothetical costs and benefits of coccolithophore calcification in a global ocean ecological model. fig. S3. Assessment against observations of modeled coccolithophore distribution for the four tested benefits of calcification. fig. S4. Observed relationship between sinking velocity, PIC/POC ratio, coccosphere size, and cell density of *E. huxleyi* (black circles) and *G. oceanica* cultured at 15°C (blue squares) and 20°C (red triangles).
