# Supplementary material for: Why marine phytoplankton calcify
Source: Sci Adv. 2016 Jul 13;2(7):e1501822. doi: 10.1126/sciadv.1501822 (PMC4956192; doi:10.1126/sciadv.1501822)
Supplement: http://advances.sciencemag.org/cgi/content/full/2/7/e1501822/DC1 [file supp_2_7_e1501822__index.html]

Science Advances | Science Advances

## Supplementary Materials

**This PDF file includes:**

- Supplementary Text
- table S1. Definition of the scores for the model-data comparison.
- fig. S1. Latitudinal biomass of two main coccolithophore types along the AMT.
- fig. S2. Testing of hypothetical costs and benefits of coccolithophore calcification in a global ocean ecological model.
- fig. S3. Assessment against observations of modeled coccolithophore distribution for the four tested benefits of calcification.
- fig. S4. Observed relationship between sinking velocity, PIC/POC ratio, coccosphere size, and cell density of *E. huxleyi* (black circles) and *G. oceanica* cultured at 15°C (blue squares) and 20°C (red triangles).

Download PDF

**Files in this Data Supplement:**

- Adobe PDF - 1501822\_SM.pdf
