# Supplementary material for: Why marine phytoplankton calcify
Source: Sci Adv. 2016 Jul 13;2(7):e1501822. doi: 10.1126/sciadv.1501822 (PMC4956192; doi:10.1126/sciadv.1501822)

Science Advances

AAAS

advances.sciencemag.org/cgi/content/full/2/7/e1501822/DC1

Supplementary Materials for

Why marine phytoplankton calcify

Fanny M. Monteiro, Lennart T. Bach, Colin Brownlee, Paul Bown, Rosalind E. M. Rickaby, Alex J. Poulton, Toby Tyrrell, Luc Beaufort, Stephanie Dutkiewicz, Samantha Gibbs, Magdalena A. Gutowska, Renee Lee, Ulf Riebesell, Jeremy Young, Andy Ridgwell

Published 13 July 2016, *Sci. Adv.* **2**, e1501822 (2016)
DOI: 10.1126/sciadv.1501822

This PDF file includes:

- Supplementary Text
- table S1. Definition of the scores for the model-data comparison.
- fig. S1. Latitudinal biomass of two main coccolithophore types along the AMT.
- fig. S2. Testing of hypothetical costs and benefits of coccolithophore calcification in a global ocean ecological model.
- fig. S3. Assessment against observations of modeled coccolithophore distribution for the four tested benefits of calcification.
- fig. S4. Observed relationship between sinking velocity, PIC/POC ratio, coccosphere size, and cell density of *E. huxleyi* (black circles) and *G. oceanica* cultured at 15°C (blue squares) and 20°C (red triangles).

Supplementary Materials

Supplementary Text

<u>Testing coccolithophore calcification trade-offs in silico</u>

Evidence for a large energetic cost associated with coccolith production is clear. However, the ecological benefit, or combination of ecological benefits coccolithophores as compared to similar non-calcifying phytoplankton, is uncertain. Indeed, the benefit (or combination of benefits) may vary between species and between different environmental regimes, greatly complicating the extrapolation of in vitro experiments to large scales and dynamic ocean environments. Here, we devise a novel approach to help test costs versus benefits of calcification as well as providing a link between the use of numerical models and oceanographic observations. The model setup is described in the Materials and Methods section of the manuscript.

In the most realistic simulations, the model diatom biomass has very similar trend to that observed along the AMT, with high values in the high latitudes and equatorial regions and lower values in the South subtropical gyre. The low subtropical value is however slightly too low (10^{-4} mg C m^{-3}) but is close to the minimum diatom biomass observed in the region (10^{-3} mg C m^{-3}). This is a common problem to biogeochemical models with similar horizontal resolution, that tend to underestimate biomass and primary production in the subtropical gyres. The model is thus able to capture the latitudinal AMT distribution of diatoms but the low spatial variability in coccolithophores biomass is not captured in any of the model simulations irrespectively of the hypothetical benefits (fig. S3). However various combinations of cost-benefits capture the diatom/coccolithophore distribution for some portion of the transect (fig S3). The model results suggest then that there is no single “benefit” of calcification, and that different reasons for calcification are important in some regions and not others. Each benefit has a distinct distribution of coccolithophores. Light uptake benefit favors coccolithophores in two areas: upwelling regions (where coccolithophores preferentially grow at the bottom of the mixed layer); and in areas with a deep mixed layer (around the Gulf Stream and Kuroshio currents). It is in these regions where light limitation becomes most important, and the increased photosynthesis at low light of the calcifying types benefited them over their non-calcifying counterparts. Protection from viral/bacterial infection benefits coccolithophores in most oceans apart from the North Pacific subpolar gyre and the Southern Ocean, which could be related to a temperature control. Grazing protection support coccolithophores relative to the non-calcifying counterparts in the most eutrophic regions where grazing pressures are the highest. There was only a very narrow successful niche for photo-damage protection benefits (fig. S2C). Photo-damage protection is a successful strategy for coccolithophores in the model only when the energetic cost is low and the protection removes any photo-damage (10% cost and 100% benefit). However, in this trade-off space coccolithophores are too successful with unrealistically high biomass and dominating phytoplankton biomass along the AMT transect, and as such the chi-square statistics found almost no “realistic” simulations.

While no tested benefit alone could account for the whole observed distribution of coccolithophores along the AMT, a combination of benefits could capture the distribution. For instance the combination of viral/bacterial and grazing protections could result in an uniform distribution of coccolithophore biomass along the AMT. Thus, the model results suggest a multi-functionality of calcification. Such a result is bolstered by the observed diversity and distribution of coccolithophores in the ocean, where placolith-bearing coccolithophores dominate in the subpolar regions (suggesting a function of grazing protection and depending on the location of light uptake and virus/bacteria protection), and where *Umbellosphaera* and *Discosphaera* grow preferentially in the subtropical regions (suggesting mostly a function of viral/bacterial protection) (fig. S3). Similarly, the observed higher diversity of

coccolithophores in the equatorial regions *(112)* compares well to the wide range of benefits suggested as successful in these regions from the model results (Fig. 4).

The realistic trade-off space for calcification in the model is associated with a large range of energetic costs (10-50% of photosynthetic cost, fig. S2). Viral/bacterial protection could provide a realistic benefit at high energetic costs (50%), however, the cost of protection from light uptake and grazing protection was only found to be beneficial for relatively low (~10%) energy cost. The amount of energy spent for calcification therefore reflects the type of benefit, suggesting physiological and/or ecological constraints. In addition, the highest costs could only support the most positive benefits, suggesting that coccolithophores calcifying at high energetic cost (e.g. heavily calcified *Coccolithus pelagicus*) can only be successful with an associated higher degree of benefit. This new modeling approach highlights that the trade-off between cost and benefit of calcification is species- and location-specific and an indicator of the adaptation of coccolithophores to their environment.

table S1. Definition of the scores for the model-data comparison. The model and observation of biomass are averaged on a 5° latitudinal band before calculating the chi-squared fit to remove some of the noise in the observations. To take into consideration the large uncertainty (or natural variability) in the observations, we also calculate a model-data error (log(P_i) - log(O_i)) by comparing with the minimum and maximum value of the observations following Harmel and Smith *(121)*'s methodology.

Name	Equation		Description
Log-transformed chi-squared fit	χ^2	$= \sum_{i=i^{min}}^{i^{max}} \frac{[\log(P_i)-\log(O_i)]^2}{\log(\sigma_i)^2}$	P is the model result; O is the observation value; i represents a 5° latitudinal band within a range of 50°N-50°S for $i^{min} - i^{max}$ values.
Model-data error	$\log(P_i) - \log(O_i)$	$= 0$ if $\log(P_i) \in [O_i^{min} - O_i^{max}]$<br>$= \log(P_i) - \log(O_i^{min})$ if $P_i < O_i^{min}$<br>$= \log(P_i) - \log(O_i^{max})$ if $P_i > O_i^{max}$	O_i^{min} is the minimum value and O_i^{max} the maximum value of the observation at the latitude band i.
Cost function	J	$= \frac{1}{2}\left(\chi^2_{Diat} + \chi^2_{Cocco}\right)$	*Diat* is for diatom and *Cocco* for coccolithophore.

Table S1

fig. S1. Latitudinal biomass of two main coccolithophore types along the AMT (combining cruises of AMT1, 2, 3, 4, 5 and 7). The observations have been averaged on a 5°-degree band. Continuous line represents the median of the observations and the dashed lines the range of observations (when none, the min observations is zero). The placolith-bearing coccolithophore species (green) include *Emiliania huxleyi*, *Gephyrocapsa* spp., *Calcidiscus leptoporus*, *Coccolithus pelagicus* and < 5μm coccospheres. The oligotrophic coccolithophore species (purple) include *Umbellosphaera* spp., *Discosphaera tubifera* and *Papposphaera lepida*. The transect shows dominance of placolith-bearing species in equatorial and mid-latitudes along the AMT and of oligotrophic species in the subtropical regions.

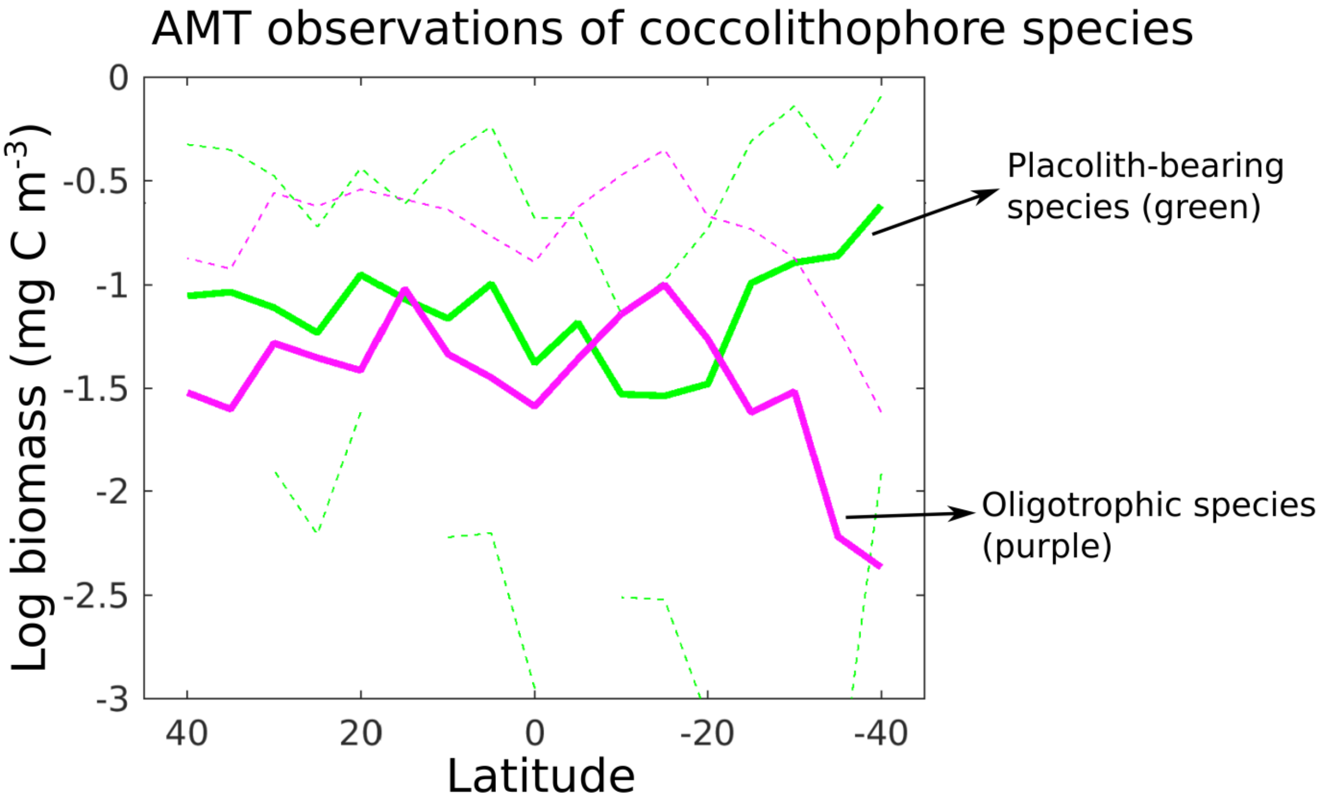

fig. S2. Testing of hypothetical costs and benefits of coccolithophore calcification in a global ocean ecological model. (**A**) Illustration of the trade-off space for testing benefits of calcification in the MITgcm ecosystem model. Crosses indicate individual model simulations with associated ecological benefit and energetic cost of calcification for the modeled Coccolithophore type. Energetic cost is represented in the model by a reduction in the maximum growth rate of the Coccolithophore type (between 10% to 90% reduction relative to non-calcifying Other large phytoplankton), while ecological benefit accounts for either (**B**) light uptake, (**C**) photo-damage protection, (**D**) viral/bacterial protection or (**E**) grazing protection (with accordingly 10% to 90% increase in benefit, see text for details). The blue area indicates simulations where coccolithophores survive. Orange area indicates simulations where the biomass of coccolithophores is comparable to observations along the AMT transect. The cost function (model-data misfit cost) is highlighted in parenthesis for each model simulation.

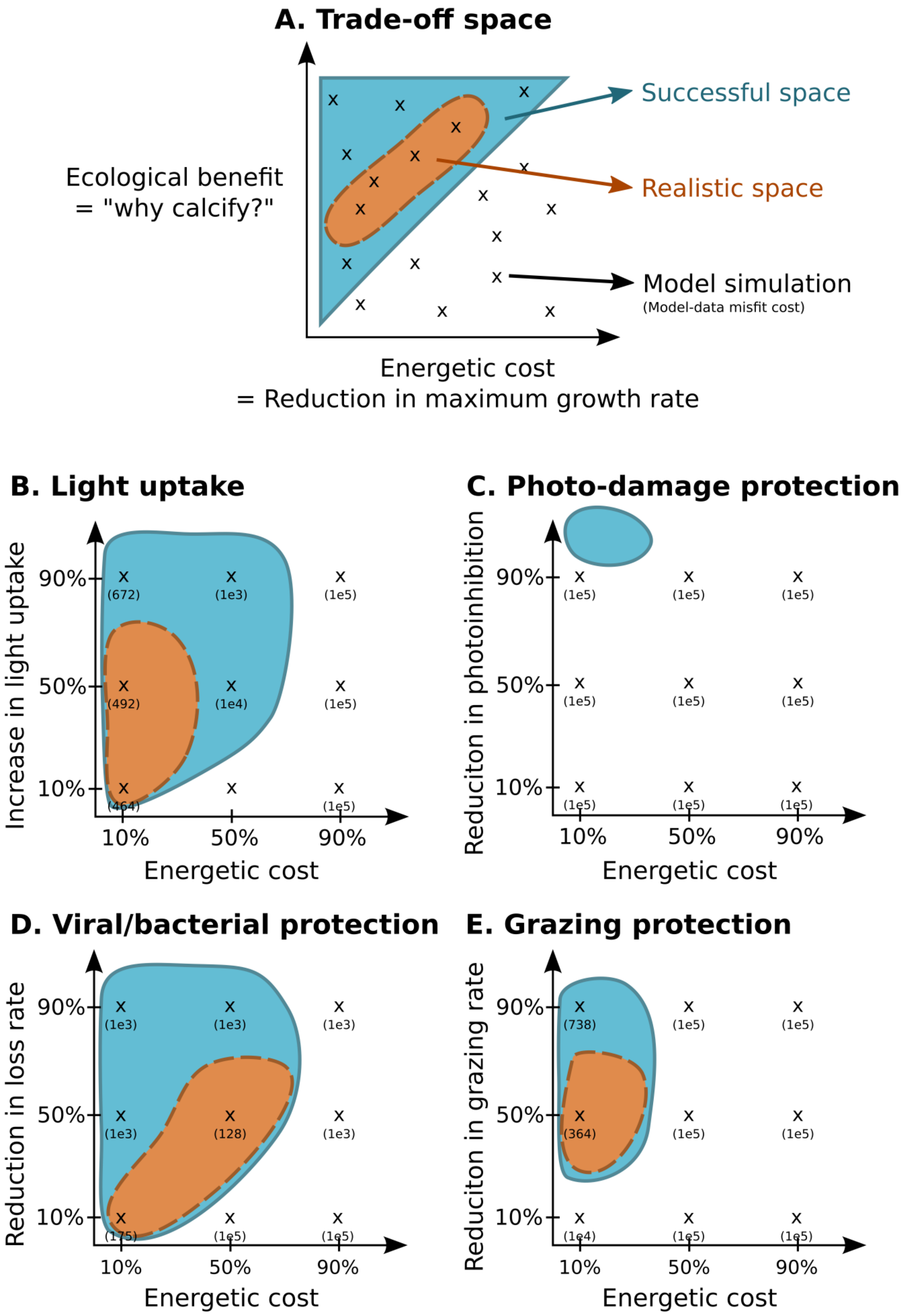
A. Trade-off space
Ecological benefit
= "why calcify?"
Successful space
Realistic space
Model simulation
(Model-data misfit cost)
Energetic cost
= Reduction in maximum growth rate
B. Light uptake
Increase in light uptake
90%
50%
10%
(672)
(1e3)
(1e5)
(492)
(1e4)
(1e5)
(464)
(1e5)
10%
50%
90%
Energetic cost
C. Photo-damage protection
Reduciton in photoinhibition
(1e5)
Energetic cost
D. Viral/bacterial protection
Reduction in loss rate
(1e3)
(128)
(175)
(1e5)
Energetic cost
E. Grazing protection
Reduciton in grazing rate
(738)
(364)
(1e4)
(1e5)
Energetic cost

fig. S3. Assessment against observations of modeled coccolithophore distribution for the four tested benefits of calcification. (**A**) Observations of biomass of coccolithophores (blue) and diatoms (red) along the Atlantic Meridional Transect (AMT) based on Cermeño *et al. (108)*. We averaged the observations on a 5° latitude band. The continuous lines represent the median value of the observations and dashed lines the range of the observations. (**B**-**E**) Most realistic model simulations of coccolithophore and diatom biomass for each tested benefit: Light uptake, photo-damage, viral/bacterial and grazing protections respectively. We selected the most realistic model simulations as best model match of combined coccolithophore and diatom biomass with the AMT observations presented in A. The cost function is presented in the text. The value of percentage benefit and cost is indicated on the top left corner of each simulation (e.g. the grazing protection most realistic simulation is for a 90% reduction of palatability and a 50% reduction of maximum growth rate).

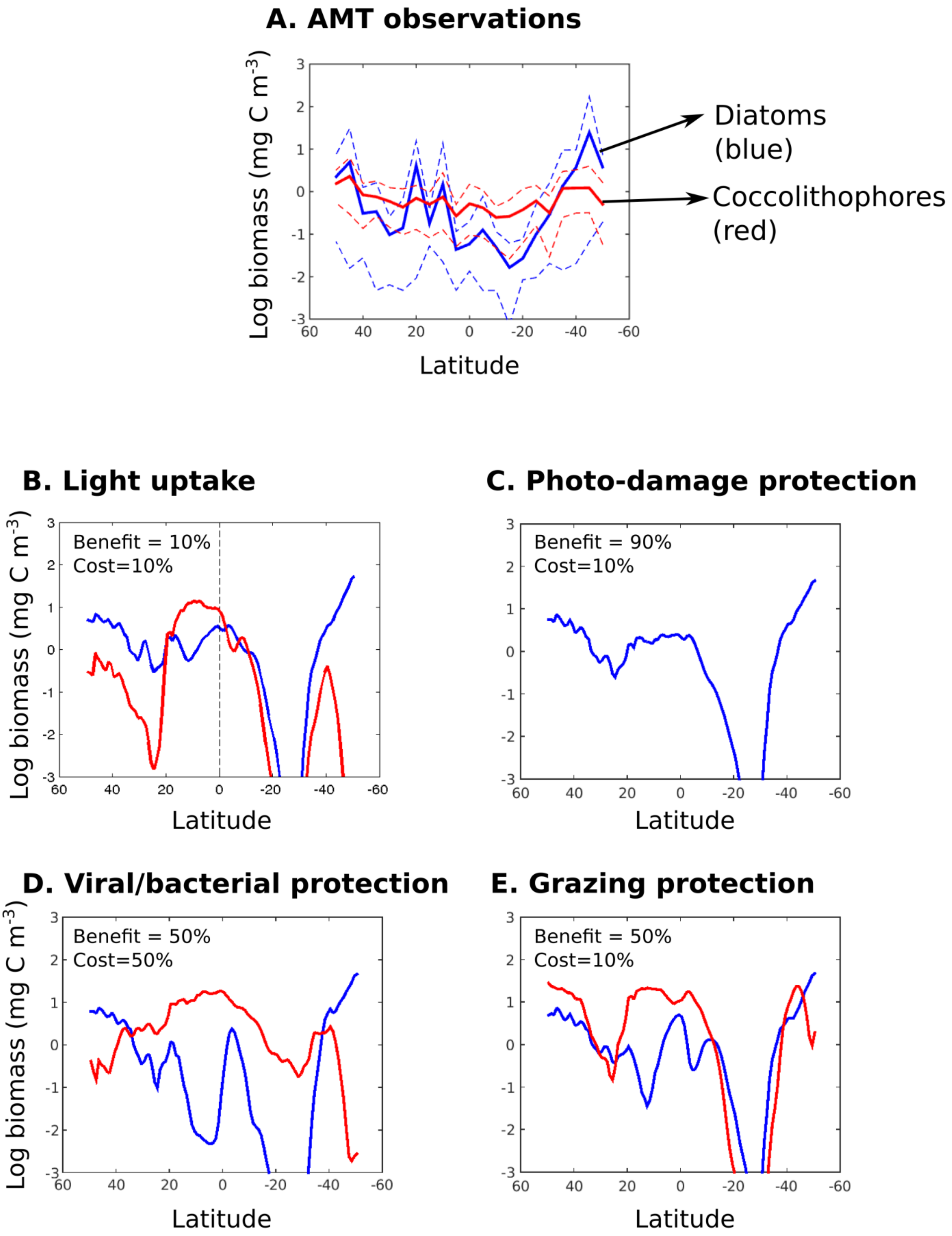
A. AMT observations
Log biomass (mg C m-3)
Latitude
Diatoms (blue)
Coccolithophores (red)
B. Light uptake
Benefit = 10%
Cost=10%
C. Photo-damage protection
Benefit = 90%
Cost=10%
D. Viral/bacterial protection
Benefit = 50%
Cost=50%
E. Grazing protection
Benefit = 50%
Cost=10%
Log biomass (mg C m-3)
Latitude

fig. S4. Observed relationship between sinking velocity, PIC/POC ratio, coccosphere size, and cell density of *E. huxleyi* (black circles), *G. oceanica* cultured at 15°C (blue squares) and 20°C (red triangles). R^2 is the coefficient of determination of the linear regression shown on top of the data. All p-values are <0.0001 meaning that all correlations are highly significant.

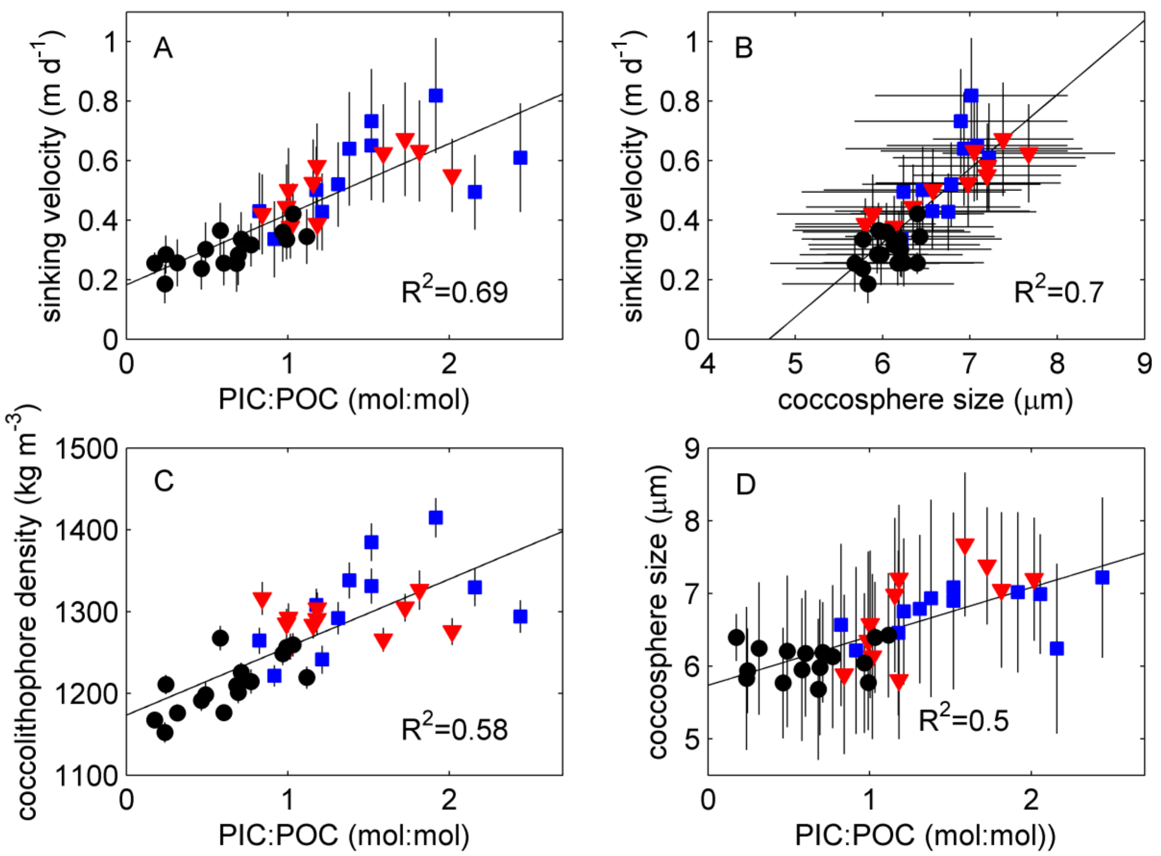

Supplement: http://advances.sciencemag.org/cgi/content/full/2/7/e1501822/DC1 [file 1501822_SM.pdf]
